# A prospective observational description of frequency and timing of antenatal care attendance and coverage of selected interventions from sites in Argentina, Guatemala, India, Kenya, Pakistan and Zambia

**DOI:** 10.1186/1742-4755-12-S2-S12

**Published:** 2015-06-08

**Authors:** Sherri Bucher, Irene Marete, Constance Tenge, Edward A Liechty, Fabian Esamai, Archana Patel, Shivaprasad S Goudar, Bhalchandra Kodkany, Ana Garces, Elwyn Chomba, Fernando Althabe, Mabel Barreuta, Omrana Pasha, Patricia Hibberd, Richard J Derman, Kevin Otieno, K Michael Hambidge, Nancy F Krebs, Waldemar A Carlo, Carolyne Chemweno, Robert L Goldenberg, Elizabeth M McClure, Janet L Moore, Dennis D Wallace, Sarah Saleem, Marion Koso-Thomas

**Affiliations:** 1 Indiana University School of Medicine, Department of Pediatrics, Indianapolis, IN, USA; 2 Moi University School of Medicine, Child Health and Paediatrics, Eldoret, Kenya; 3 Indira Gandhi Government Medical College; Lata Medical Research Foundation, Nagpur, India; 4 KLE University’s Jawaharlal Nehru Medical College, Belgaum, India; 5 Fundación para la Alimentación y Nutrición de Centro América y Panamá, Guatemala City, Guatemala; 6 University Teaching Hospital, Lusaka, Zambia; 7 Institute for Clinical Effectiveness and Health Policy, Buenos Aires, Argentina; 8 Aga Khan University, Karachi, Pakistan; 9 Massachusetts General Hospital, Boston, MA, USA; 10 Christiana Care Health Services, Newark, DE, USA; 11 University of Denver School of Medicine, Denver, CO, USA; 12 University of Alabama at Birmingham, Birmingham, AL, USA; 13 Department of Obstetrics/Gynecology, Columbia University, New York, NY, USA; 14 RTI International, Durham, NC, USA; 15 Eunice Kennedy Shriver National Institute of Child Health and Human Development, Bethesda, MD, USA

**Keywords:** Maternal-newborn health; birth registry; antenatal care; Africa; Asia; Latin America; focused antenatal care; quality of care

## Abstract

**Background:**

The Global Network for Women’s and Children’s Health Research is one of the largest international networks for testing and generating evidence-based recommendations for improvement of maternal-child health in resource-limited settings. Since 2009, Global Network sites in six low and middle-income countries have collected information on antenatal care practices, which are important as indicators of care and have implications for programs to improve maternal and child health. We sought to: (1) describe the quantity of antenatal care attendance over a four-year period; and (2) explore the quality of coverage for selected preventative, screening, and birth preparedness components.

**Methods:**

The Maternal Newborn Health Registry (MNHR) is a prospective, population-based birth and pregnancy outcomes registry in Global Network sites, including: Argentina, Guatemala, India (Belgaum and Nagpur), Kenya, Pakistan, and Zambia. MNHR data from these sites were prospectively collected from January 1, 2010 – December 31, 2013 and analyzed for indicators related to quantity and patterns of ANC and coverage of key elements of recommended focused antenatal care. Descriptive statistics were generated overall by global region (Africa, Asia, and Latin America), and for each individual site.

**Results:**

Overall, 96% of women reported at least one antenatal care visit. Indian sites demonstrated the highest percentage of women who initiated antenatal care during the first trimester. Women from the Latin American and Indian sites reported the highest number of at least 4 visits. Overall, 88% of women received tetanus toxoid. Only about half of all women reported having been screened for syphilis (49%) or anemia (50%). Rates of HIV testing were above 95% in the Argentina, African, and Indian sites. The Pakistan site demonstrated relatively high rates for birth preparation, but for most other preventative and screening interventions, posted lower coverage rates as compared to other Global Network sites.

**Conclusions:**

Results from our large, prospective, population-based observational study contribute important insight into regional and site-specific patterns for antenatal care access and coverage. Our findings indicate a quality and coverage gap in antenatal care services, particularly in regards to syphilis and hemoglobin screening. We have identified site-specific gaps in access to, and delivery of, antenatal care services that can be targeted for improvement in future research and implementation efforts.

**Trial registration:**

Registration at Clinicaltrials.gov (ID# NCT01073475)

## Background

The World Health Organization (WHO) recognizes the importance of antenatal care (ANC) within a continuum of reproductive and maternal-newborn care. WHO recommends early identification of pregnancy and at least four ANC visits, starting prior to 14 weeks gestation, during which a prescribed package of preventative, screening, and educational interventions are delivered [1]. Unfortunately, such a consistent, integrated continuum of comprehensive care—although defined on paper, and codified in many national health policies—often does not translate into actual practice in some resource-limited settings [2]. For example, while it is widely acknowledged that ANC is most effective if it is initiated early and consistently throughout pregnancy[3], many pregnant women in resource-limited settings (1) initiate ANC late in pregnancy (e.g., during the 2^nd^ or 3^rd^ trimester) and (2) do not receive the WHO-defined minimum of at least 4 ANC visits. In addition to the frequency and timing of ANC visits, the quality of ANC care, as assessed by comprehensive, effective coverage of key evidence-based elements of the WHO Focused Antenatal Care (FANC) package, is also of concern [4-9]. Although the strength of the evidence-base for some components of the focused ANC package is variable, FANC as a bundled, integrated intervention can serve as an important pathway by which to increase the probability that women will chose facility-based births with skilled delivery attendants, versus home births with family members or unskilled attendants [10-12]. It is important to delineate the gaps, barriers, and facilitators which impact access to comprehensive coverage of recommended ANC interventions, as this has profound implications for improving the entire continuum of maternal newborn child health (MNCH) care in low-resource settings.

Currently, the Global Network for Women’s and Children’s Health, funded by the National Institute of Child Health and Human Development (NICHD), is one of the largest global research networks focused exclusively on testing and generating evidence-based recommendations for improvement of maternal-child health in resource-limited settings [13]. Given the importance of an integrated ANC continuum toward positive health outcomes among our populations of mothers and babies, to assess the quality of ANC coverage among Global Network sites, and to identify relevant targets for future research and implementation initiatives, we sought to: (1) describe the quantity (i.e., number of visits; timing of ANC initiation) of ANC attendance and (2) quality of ANC attendance in terms of coverage of key preventative, screening, and birth preparedness components.

## Methods

The Maternal Newborn Health Registry (MNHR) is a prospective, population-based birth and pregnancy outcomes registry that was started in 2009 at communities in seven Global Network sites, including: Argentina, Guatemala, India (Belgaum and Nagpur), Kenya, Pakistan, and Zambia. A detailed description of the overall purpose, methods, and data collection techniques for the Global Network for Women’s and Children’s Health Research Maternal Newborn Health MNHR is provided elsewhere [13-15].

For the current study, MNHR data from 106 clusters for the period January 1, 2010 – December 31, 2013 were analyzed for variables related to the patterns of ANC (e.g., number and timing of ANC visits; where, and from whom, ANC was obtained) and coverage of selected elements of the WHO FANC package.

Data were collected and entered at each study site and transmitted through secure methods to a central data coordinating center at Research Triangle Institute International (RTI). Data edits were performed at each study site and then centrally. Descriptive statistics were generated overall, by global region (sub-Saharan Africa, South Asia, and Latin America), and for each individual site. In order to investigate the numbers and percentages of women (overall, by region, and by site) who reported having components of a birth/emergency preparedness plan, we also explored a sub-set of data which were collected during the course of a trial for implementation of community-based Emergency Obstetric and Neonatal Care (EmONC) [16]. Because the EmONC study intervention may have influenced results from the intervention clusters, for the purposes of the current study, we report the results from the women enrolled only in the control clusters. All analyses were performed using SAS version 9.3 (SAS Institute, Cary, NC, USA).

In order to present a more comprehensive assessment of the quality/quantity of ANC by site, we developed a composite variable composed of 15 ANC indicators. The composite variable allows a comparison of how each Global Network site complied with a variety of ANC indicators, which, when assessed collectively, provide an indication, within and between sites, of the rate of coverage for bundled ANC service delivery. For each indicator we arbitrarily grouped compliances and categorized these as good, fair or poor. As an example, for having at least one ANC visit, good was classified as >95%, fair as 85-94% and poor as <85%. Good, fair and poor for tetanus toxoid, provision of iron and vitamins used the same percentage categories. For all the other indicators, good was classified as >75%, fair as 50-75% and poor<50%. These included: having at least four ANC visits, syphilis testing; HIV testing; anemia testing; maternal height measured; maternal weight measured; birth plan developed; transport plan developed; birth attendant identified, and identified birth attendant present at delivery. We then simply compiled the number of good, fair and poor answers for each site to develop a figure to illustrate these metrics. Because the Argentina site expected virtually all women to deliver in a hospital, we coded the categories of birth plan developed; transport plan developed; birth attendant identified, and identified birth attendant present at delivery as good for this site.

## Ethical review

The appropriate Institutional Review Boards and Ethics Research Committees of the participating institutions and the Ministries of Health of the respective countries approved the MNHR. Prior to initiation of the study, approval was sought from the participating communities through sensitization meetings. Individual informed consent for study participation is requested from each study participant. No monetary reimbursements are provided to study participants nor to the communities participating in the study. A Data Monitoring Committee, appointed by the NICHD, oversees and reviews the study at annual meetings.

## Results

**Figure 1:**
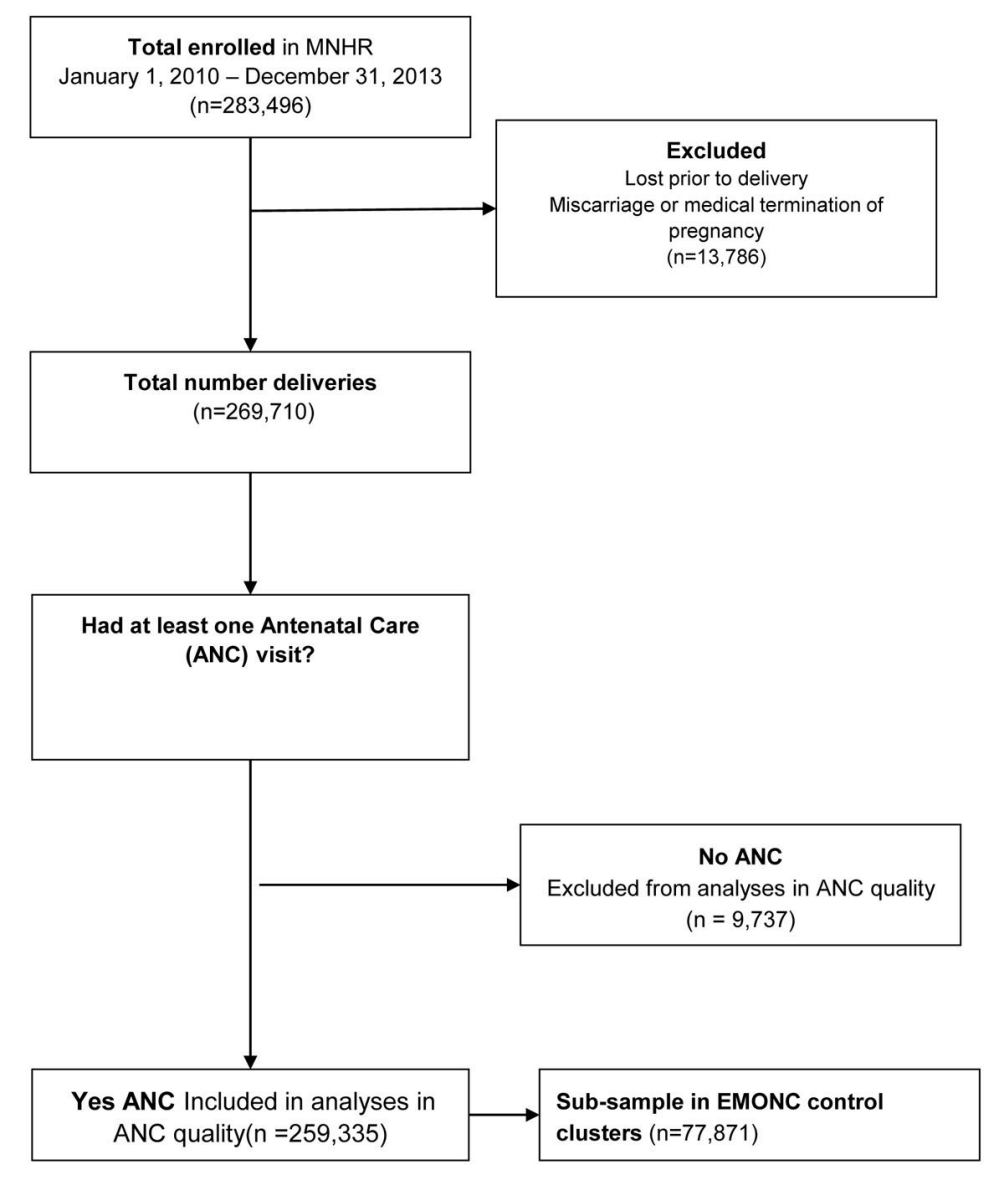
Diagram of study population

Data from a total of 269,710 deliveries (excludes women who were lost-to-follow-up prior to delivery; miscarried; or underwent medical termination of pregnancy) were used to explore the frequency of ANC (Figure 1). By global region, 23% of these births occurred in African sites, 62% in Asian sites and 15% in Latin American sites.

Number of ANC visits (Table 1): Ninety-six percent of women reported at least one ANC visit during their pregnancy. In the Nagpur and Belgaum, India sites, respectively, 100% and 99.9% of women reported at least one antenatal encounter. High rates of at least one ANC visit were also reported among pregnant women in the sites in Zambia (99%), Guatemala (98%), Kenya (97%), and Argentina (95%). The Pakistan site reported the lowest percentage (84%) of women with at least one ANC encounter.

**Table 1:** Antenatal care by Global Network region and site

	Africa		Asia			Latin America		Total
		
	Kenya	Zambia	Belgaum	Nagpur	Pakistan	Argentina	Guatemala	
Women with delivery, N	35,660	27,599	79,628	39,261	48,273	9,900	29,389	269,710

At least one ANC visit, N (%)	34,637 (97.2)	27,419 (99.4)	79,355 (99.9)	39,215 (100.0)	40,540 (84.4)	9,310 (95.0)	28,859 (98.3)	259,335 (96.4)

Number of ANC visits^1^								

0	321 (1.5)	36 (0.4)	17 (0.0)	15 (0.1)	3,139 (11.2)	144 (4.1)	319 (1.5)	3,991 (2.7)

1	977 (4.5)	692 (7.0)	1,391 (3.5)	507 (2.4)	5,438 (19.3)	185 (5.3)	612 (2.9)	9,802 (6.8)

2	3,309 (15.2)	1,901 (19.2)	2,626 (6.6)	378 (1.8)	7,508 (26.7)	287 (8.2)	1,407 (6.8)	17,416 (12.0)

3	7,880 (36.3)	5,087 (51.4)	11,758 (29.4)	3,070 (14.6)	5,369 (19.1)	568 (16.3)	2,873 (13.8)	36,605 (25.2)

> 3	9,245 (42.5)	2,181 (22.0)	24,258 (60.6)	17,088 (81.1)	6,695 (23.8)	2,300 (66.0)	15,575 (74.9)	77,342 (53.3)

Trimester for first ANC visit								

First	1,288 (3.9)	2,270 (8.4)	48,920 (63.3)	30,330 (77.5)	9,528 (24.5)	3,278 (37.4)	11,799 (41.5)	107,413 (42.5)

Second	19,700 (58.9)	19,465 (72.0)	24,055 (31.1)	7,699 (19.7)	12,654 (32.5)	3,937 (44.9)	12,538 (44.1)	100,048 (39.5)

Third	12,444 (37.2)	5,304 (19.6)	4,325 (5.6)	1,106 (2.8)	16,727 (43.0)	1,545 (17.6)	4,110 (14.4)	45,561 (18.0)

Most frequent location of ANC, N (%)								

Gov. hospital	28,795 (83.4)	7,792 (28.5)	35,886 (47.6)	14,427 (44.8)	8,297 (21.3)	7,535 (81.3)	3,503 (15.0)	106,235 (44.1)

Private hospital	494 (1.4)	21 (0.1)	20,474 (27.1)	869 (2.7)	1,833 (4.7)	22 (0.2)	396 (1.7)	24,109 (10.0)

Gov. clinic	3,870 (11.2)	8,657 (31.7)	7,900 (10.5)	5,590 (17.4)	58 (0.1)	247 (2.7)	6,688 (28.6)	33,010 (13.7)

Private clinic	465 (1.3)	902 (3.3)	710 (0.9)	58 (0.2)	28,468 (73.1)	644 (6.9)	1,519 (6.5)	32,766 (13.6)

Health worker	61 (0.2)	30 (0.1)	10,425 (13.8)	11,191 (34.8)	79 (0.2)	8 (0.1)	2,487 (10.6)	24,281 (10.1)

TBA	823 (2.4)	247 (0.9)	72 (0.1)	35 (0.1)	201 (0.5)	0 (0.0)	8,774 (37.5)	10,152 (4.2)

Other	8 (0.0)	9,663 (35.4)	3 (0.0)	22 (0.1)	11 (0.0)	816 (8.8)	54 (0.2)	10,577 (4.4)

Across all sites, 53% of women reported having at least four antenatal encounters. The Nagpur (81%), Guatemala (75%) and Argentina (66%) sites reported the highest rate of pregnant women who accessed ANC at least 4 times, with sites in Belgaum, India following at 61%. African sites (Zambia, 22%; Kenya, 43%) and the Pakistan site (24%) reported the lowest rates of pregnant women with at least 4 ANC encounters.

**Figure 2:**
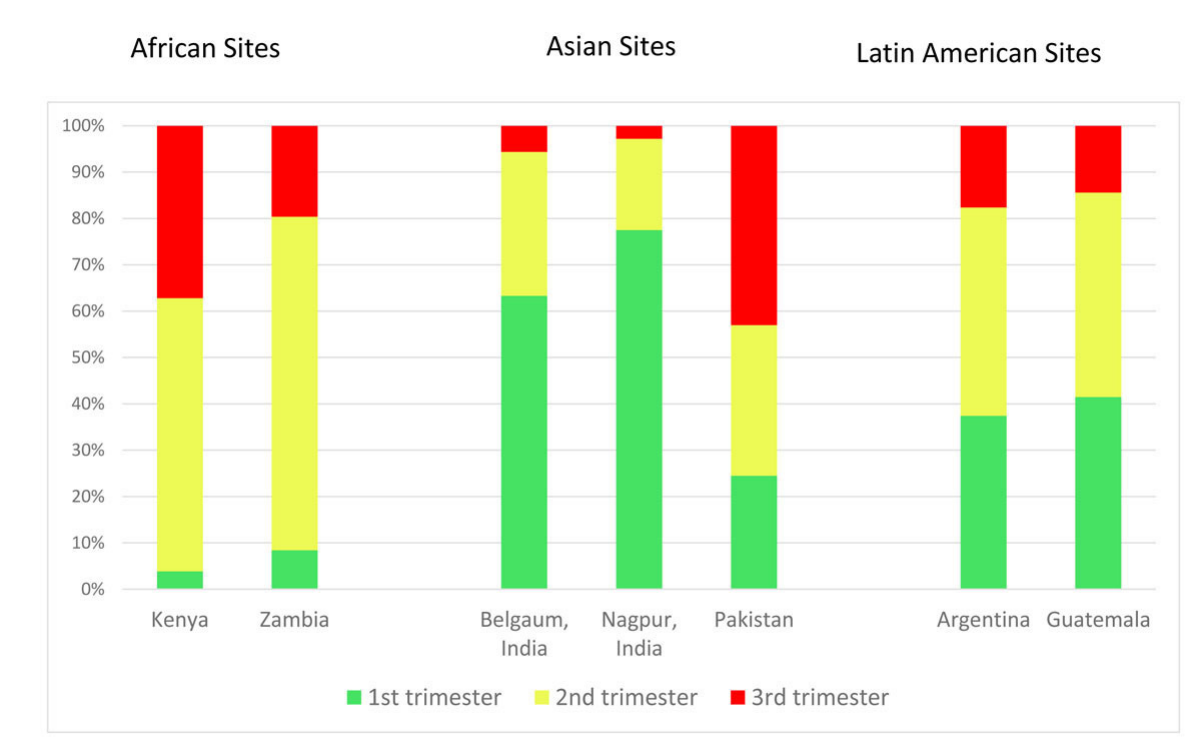
Percentage of Global Network women who initiated ANC in the first, second, and third trimesters

Timing of ANC access (Figure 2): Pregnant women from the two Indian sites were more likely to initiate ANC during the first trimester (63% for Belgaum and 78% for Nagpur, respectively), and reported the lowest percentages of initial ANC visits during the third trimester (6% and 3%). Sites in Kenya and Zambia reported the lowest initiation of ANC during the first trimester, at only 4% and 8%, respectively. The Pakistan (43%) and Kenya (37%) sites reported the highest number of women who had their first ANC encounter during the third trimester of pregnancy.

Given the high percentage of women who initiated ANC during the first trimester in the Indian sites as compared to the other sites, we investigated the overall timing of ANC initiation with and without the India data included. Overall, with the India sites included, across all Global Network sites, 43% of women began ANC during the first trimester, 40% during the second trimester, and 18% during the third trimester of pregnancy. With the Indian data excluded, among women in sites in Argentina, Guatemala, Kenya, Pakistan, and Zambia, there was a marked shift toward first ANC visits occurring in the second (50%) and third (29%) trimesters.

Location and provider where ANC obtained (Table 1): A government hospital was the most commonly reported location where women across the entire study obtained ANC (44%), particularly in the Kenyan (83%) and Argentinian sites (81%). In the Zambian site, women received their ANC from either a government hospital (29%) or clinic (32%), while 35% received their care from “others.” Women from the Belgaum, India site reported frequent use of both government (48%) and private hospitals (27%); in the Nagpur site, women most frequently selected government hospitals (45%) and health workers (35%). The most frequent location where ANC was obtained in the Pakistan site was from private clinics (73%). In the Guatemalan site, 38% of women reported receiving ANC from a traditional birth attendant (TBA) and 29% from a government clinic.

### Quality of antenatal care

The quality of ANC in regards to provision of specific preventive and screening interventions was gathered from among women who reported at least one ANC encounter (259,335). A sub-population of women who participated in a separate time-limited, cluster randomized MNHR-based study [15] (77,871, Figure 1)were further assessed for whether they had generated a birth and emergency preparedness plan.

Overall rates of coverage for all sites for tetanus toxoid vaccination was 88% (227,942) (Table 2). High rates of vaccination were observed in the Indian sites [Belgaum, 99.8%; Nagpur, 99.7%], Argentinian sites (96%), and African sites [Zambia (96%); Kenya (93%)]. Lowest coverage rates were reported in the Guatemalan (67%) and Pakistan (57%) sites. (The low rate in Guatemala is explained by a government recommendation not to revaccinate if vaccinated in the last 10 years).

**Table 2:** Coverage of various components of WHO recommendations by Global Network region and site among women who reported at least one antenatal care visit, 2010-2013

	Africa		Asia			Latin America		Total
		
	Kenya	Zambia	Belgaum	Nagpur	Pakistan	Argentina	Guatemala	
Tetanus toxoid vaccine, N (%)	32,032 (92.5)	26,273 (95.8)	79,207 (99.8)	39,111 (99.7)	23,138 (57.1)	8,871 (96.0)	19,310 (67.2)	227,942 (88.0)

								

Prenatal vitamins/iron, N (%)	32,645 (94.3)	27,115 (98.9)	78,934 (99.6)	38,928 (99.5)	29,567 (73.0)	8,222 (89.3)	26,685 (92.5)	242,096 (93.5)

								

Syphilis test**, N (%)	27,650 (79.8)	23,338 (85.1)	14,753 (18.8)	18,073 (46.7)	59 (0.1)	8,799 (95.3)	12,847 (44.8)	105,460 (48.6)

								

HIV test**, N (%)	33,403 (96.4)	26,883 (98.0)	78,412 (99.4)	38,310 (97.9)	147 (0.4)	8,747 (94.8)	13,056 (45.6)	198,811 (91.2)

								

Hemoglobin taken, N (%)	11,300 (32.6)	5,658 (20.6)	68,271 (86.0)	38,620 (98.5)	694 (1.7)	4,142 (44.5)	121 (0.4)	128,806 (49.7)

								

Maternal height taken, N (%)	11 (0.0)	27,368 (99.8)	74,064 (93.3)	39,201 (100.0)	40,338 (99.5)	6,533 (70.2)	18,566 (64.3)	206,081 (79.5)

								

Maternal weight taken, N (%)	25,393 (73.3)	27,327 (99.7)	75,077 (94.6)	39,191 (99.9)	40,327 (99.5)	8,293 (89.1)	17,929 (62.1)	233,537 (90.1)

*Numbers less than total reflect missing

**Pakistan numbers omitted from total column because testing not part of recommended ANC package

Ninety-four percent of women, overall, reported receiving prenatal vitamins/iron; coverage for prenatal vitamins/iron was above ninety percent in the Indian, Zambian, Kenyan, and Guatemalan sites, but lower in the Argentinian (89%) and Pakistan (73%) sites.

Screening for syphilis and HIV are not included in the Pakistani recommended national package of ANC services; [16] thus, coverage rates for syphilis and HIV screening are considered for the remaining countries. Overall, 49% of women across the other 6 sites reported receiving syphilis testing. The Argentina site posted the highest rate of syphilis testing at 95%, followed by the Zambian (85%) and Kenyan (80%) sites. The Belgaum, India sites (19%), reported the lowest rate of coverage for syphilis testing, followed by the sites in Guatemala (45%) and Nagpur (47%). Overall coverage for HIV testing during pregnancy was 91%. Rates were 94% or higher in the Indian, Zambian, Argentinian, and Kenyan sites. In the Guatemala site, HIV testing was only conducted in 46% of pregnancies.

Overall, 50% of women who had at least one ANC encounter reported being screened for anemia. The lowest rates of hemoglobin testing were observed in the Guatemalan (0.4%) and Pakistan sites (1.7%) and the highest reported rate in the Nagpur, India site (98.5%). The Belgaum, India site performed hemoglobin assessments in 86% of pregnancies. In African sites, only 21% and 33% of women in Zambia and Kenya, respectively, reported having a hemoglobin test; in the Argentina site, 45% of women were screened for anemia.

Information on maternal height and weight was taken for 80% and 90% of women, respectively. Very high rates maternal height and weight screening were observed in sites in Nagpur, India (100%; 99.9%), Pakistan (99.5%, 99.5%), and Zambia (99.8%, 99.7%). The Belgaum, India site assessed height in 93% of pregnant mothers and weight in 95%. Latin American sites, Argentina and Guatemala, showed overall coverage rates for maternal height of 70% and 64%, and weight, 89% and 62%, respectively. Maternal height information was available for 0% of Kenyan women. When the Kenyan site was removed from analysis, the overall rate of maternal height assessment for the remaining sites rose to 92%.

Birth plan and emergency preparedness (Table 3): A sub-sample of women (N=77,871) was assessed for four elements related to preparation of a birth plan. Overall, 68.0% of women reported having funds saved for the delivery, ranging from 87.0% in the Belgaum, India site to 43.8% in the Kenya site. A birth attendant was identified to perform the delivery for 82.5% of women overall, ranging from 97.5% for women in the Guatemala site to 50.1% for women in the Zambia site. For 70.0% of the women who reported having identified a birth attendant prior to delivery, the identified birth attendant was present at the birth. Finally, 68.5% of the women identified plans for transportation to a facility for the delivery, ranging from 95.5% of women at the Belgaum, India site to 31.0% of women in the Guatemala site.

**Table 3:** Development of a birth and emergency preparedness plan during antenatal care among women in the Global Network EmONC trial control clusters

	Africa		Asia			Latin America	Total
		
	Kenya	Zambia	Belgaum	Nagpur	Pakistan	Guatemala	
Women with delivery in control cluster, N	11,453	9,600	19,053	12,710	16,053	5,754	77,871

							

Access to emergency fund/plan for hospitalization, N (%)	5,011 (43.8)	5,810 (60.8)	16,320 (87.0)	10,522 (83.1)	12,061 (75.4)	2,801 (48.7)	52,668 (68.0)

							

Birth attendant identified before birth, N (%)	9,391 (82.0)	4,794 (50.1)	17,000 (89.3)	10,844 (85.4)	13,815 (86.3)	5,595 (97.5)	61,439 (82.5)

							

Identified birth attendant at delivery N (%)	7,897 (84.1)	3,684 (86.2)	11,174 (65.7)	5,944 (54.8)	9,321 (67.5)	4,607 (82.3)	42,627 (70.0)

							

Transport identified prior to delivery, N (%)	5,541 (48.4)	4,060 (42.6)	17,933 (95.5)	10,444 (82.7)	11,043 (69.0)	1,781 (31.0)	50,802 (68.5)

**Figure 3:**
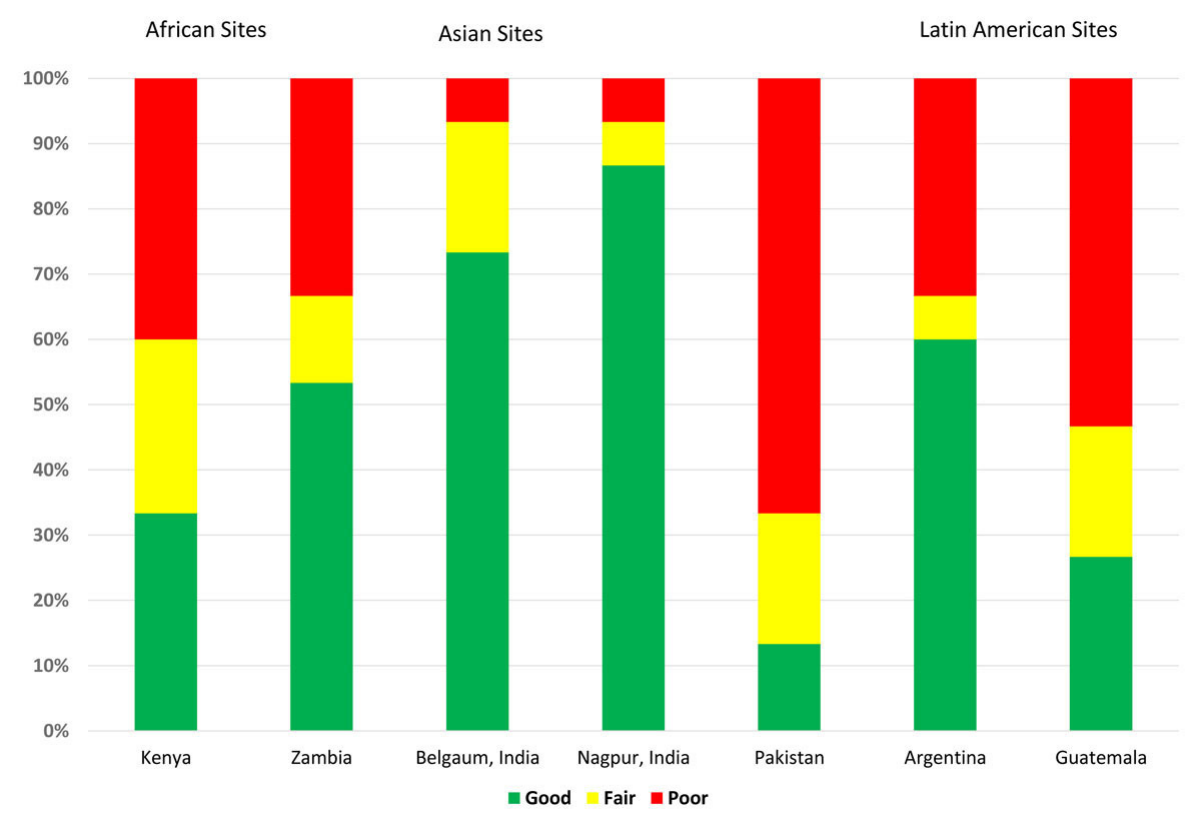
Ranking of “good,” “fair,” and “poor” coverage by Global Network sites for 15 WHO ANC indicators of access and quality of care

Composite variable to assess overall quality of ANC coverage (Figure 3): In order to present a more comprehensive assessment of the quality of ANC by site, we developed a composite variable composed of the 15 ANC WHO indicators. In this analysis, the Indian sites graded the highest, while the sites in Kenya, Zambia, Argentina and Guatemala were in the middle; the Pakistan site graded the worst. However, at every site there was room for improvement in at least some of the indicators of antenatal care.

## Discussion

### Frequency, timing, location, and providers of antenatal care

Almost all women reported accessing ANC at least once. Women in the Indian sites reported initiation of ANC during the first trimester much more frequently as compared to the other GN sites. Our study revealed differences between regions and sites in terms of from whom, and from where, women sought ANC. While a variety of complex sociocultural, behavioral, and contextual factors impact women’s decision-making in regards to when to initiate, and how often to seek, ANC [8,11,12,17-32], it is interesting to note that, in the Guatemalan site, where 38% of women reported using TBAs for ANC, 75% of women reported at least four ANC encounters and 42% of women initiated ANC during the first trimester. This is in contrast to GN sites in Africa and Pakistan, where women more frequently utilized government facilities (Kenya and Zambia) or private clinics (Pakistan) and also initiated ANC much later in pregnancy and reported fewer ANC encounters overall. Future studies in our Network should further explore the complex relationships between, and identify the facilitators and barriers which underlie, women’s decision-making in regards to frequency, timing, location, and providers of ANC. Such investigations may provide information by which stakeholders in low- and middle-income settings can improve ANC access and address gaps in coverage of key interventions.

### Coverage of preventative and screening interventions

In many low- and middle-income settings, recommended packages of ANC are already standard of care according to national policy, but recommended evidence-based interventions still fail to reach every pregnant woman [3,4,33,34]. We investigated rates of coverage for ANC interventions related to prevention, screening, and birth preparedness among our GN sites.

Detection, prevention, and treatment of syphilis may be one of the highest impact interventions available for prevention of stillbirth;[35,36] as such, syphilis screening during pregnancy is a key intervention for prevention of poor pregnancy outcomes [37-40]. It was thus discouraging to find that, only about half of all women (excluding Pakistan) reported having been tested for syphilis during their most recent pregnancy. Results from the site in Belgaum, India, were particularly interesting, in that the low rates of reported syphilis testing (only 19% of women) were atypical as compared to this site’s high performance on other ANC indicators. While this may, in part, reflect dropping prevalence of maternal and congenital syphilis within the Indian setting [37], it does not entirely explain why syphilis screening rates were much higher in the site in Nagpur, India compared to the Belgaum, India site.

HIV screening during pregnancy is essential in order to identify HIV-infected women, and link them to strategies for prevention of mother-to-child transmission of HIV [41]. It was encouraging to observe high rates of HIV testing overall, with the exception of the Guatemalan site, in which only a little over half of all women reported being tested for HIV.

Neonatal tetanus also remains a profound risk to newborn health in resource-limited regions, particularly sub-Saharan Africa, and is best prevented by maternal tetanus immunization [42-44]. The rate for at least one injection of tetanus toxoid was 88% for the GN overall, with very high coverage rates (above 93%) observed in the sites in Africa, India, and Argentina. Similar to other indicators, low rates of coverage for this preventative intervention were observed in the Pakistan site [45,46]. We did not collect information regarding how many doses of tetanus toxoid women received, so it is unknown to what extent there was complete coverage for prevention of maternal/neonatal tetanus at the GN sites, even among the 88% of pregnant women who reported receiving an injection.

Anemia during pregnancy is related to a number of poor pregnancy outcomes for mothers and newborns, and of particular concern in malarial endemic areas of the world [47,48]; thus, hemoglobin assessment and supplementation during pregnancy with prenatal vitamins and iron are key ANC recommendations at all GN sites [43]. We detected large variability in rates of screening for anemia, with coverage ranging from virtually none in the sites in Guatemala and Pakistan to nearly 100% in Nagpur, India. In Kenya and Zambia, where malaria, helminthic infection, and iron-deficiency anemia are prevalent, [47,49,50] lower than desired levels of coverage for hemoglobin testing were observed. Encouragingly, the vast majority of women did report obtaining prenatal vitamins/iron supplementation during pregnancy. We did not collect information regarding for how long, or when, women took prenatal vitamins/iron, so it is not possible to assess whether women received 90 days of iron-folate supplementation as is recommended in some contexts [51] or if the consumption of prenatal vitamins/iron was continuous, intermittent, or some combination thereof [52].

Variable rates of syphilis and HIV screening, hemoglobin testing, and tetanus toxoid vaccination that were observed in this study may, in part, reflect different priorities of national governments, based on variable prevalence rates of sexually transmitted infections among pregnant women in different global regions [44,53-55], or be influenced by the relative availability of resources for immunization and/or blood testing (education and training of health workers; community sensitization; supply chain logistics) [56,57]. Cultural barriers, fear, stigma, and resistance to some interventions in general, or among pregnant women in particular, also exist in some settings, and has been cited as impacting screening rates for sexually transmitted infections and tetanus toxoid vaccination efforts [58]. Other contributing factors to site or regional differences for coverage of particular interventions, including iron/folate supplements, may include: lack of knowledge among pregnant women as to the deleterious health impact and/or importance of screening for conditions such as syphilis, tetanus, or anemia [29,59,60]; late initiation of, and infrequent utilization of ANC; and perceived or actual financial barriers associated with obtaining interventions [61].

It is unclear if, or in what manner, the selected location and/or provider for ANC may have impacted quality of ANC, or contributed to gaps in coverage of key interventions. However, in our study, 73% of women in the Pakistani site reported using private clinics for ANC. Majrooh and colleagues recently noted that in Pakistan, private clinics, in particular, “are unable to provide the essential laboratory services package included in the standard ANC protocol,” [8]. Similarly, 38% of Guatemalan women reported seeking ANC from TBAs. Within the Guatemalan setting, TBAs are generally not equipped with the knowledge, skills, or materials to perform blood-based testing procedures, nor do they have access to laboratory support [62]. Lack of equipment was likely one key factor underlying poor coverage for maternal height assessment in Kenya, as most government facilities do not have stadiometers.

For the Argentina site in particular, higher rates of tetanus toxoid vaccination, prenatal vitamin supplementation, and syphilis and HIV testing may be due in part to women desiring technically advanced ANC, and actively seeking medical interventions from well-equipped government facilities [63]. Reasons for less than half of Argentinian women receiving hemoglobin screening are less clear.

### Birth preparedness

Access to skilled birth attendants and care during delivery saves maternal and newborn lives [64]; preparing a birth plan and planning for emergencies during the antenatal period can improve women’s willingness to seek skilled care at delivery [65]. We assessed the frequency with which women: (a) reported having prepared an emergency/ hospitalization fund; (b) identified transport options prior to delivery; (c) identified a birth attendant prior to delivery; and (d) whether or not the identified birth attendant was present at the delivery. In general, for the first three birth plan components, the Asian sites (India and Pakistan) reported the highest coverage levels. The Guatemalan and Zambian sites reported the highest numbers of women for whom the birth attendant they identified prior to delivery actually attended the birth. In Guatemala, this result may be linked with a greater proportion of women also receiving care from TBAs. In Zambia, while 60% of respondents most frequently sought ANC at a government hospital or clinic, 35% of women received ANC from “other” (not TBA). It is unclear if, or how, this may have impacted patterns of ANC access and coverage of ANC interventions.

Because of the large quantity of data regarding the quality indicators, we developed a simple grading system to provide an overall site assessment regarding the delivery of a comprehensive package of ANC at each location. We realize the categories used and the cutoffs for classification are arbitrary. Nevertheless, Figure 3 provides a visual assessment of the quality/quantity of ANC at the individual sites. The overall poor result for Pakistan in particular may explain, in part very high mortality rates reported at this site in another paper in this series [66]. We hope that by assessing the quality/quantity of both: (a) individual ANC components and (b) delivery of the overall ANC package within and between sites, this will assist stakeholders to identify targets which lead to improvements in both individual and bundled ANC indicators over time.

The strengths of our study include the population based prospective nature of the data collection, large sample size, and the quality of the MNHR in regards to high consent rates, low loss-to-follow up rates, and overall data quality [13-15,67]. We collected information about a wide variety of global indicators for ANC access and coverage. However, some ANC indicators of specific regional interest, such as those related to detection and prevention of malaria in pregnancy, were not assessed.

Limitations include that ANC registry data are collected primarily through maternal or birth attendant report and may reflect recall bias. Some overall results should be interpreted cautiously, since a large proportion of our sample is from the study sites in Asia, as compared to other global regions which compose the MNHR. Our study catchment areas are predominately rural clusters in confined regions of six countries, and as such, the results may not be representative of patterns from urban settings, in particular, or entire national or global regions, in general.

However, this latter concern is somewhat attenuated by the fact that, for the most part, our results align with those reported from recent National Household and/or Demographic and Health Surveys (DHS) [46]. One strength of our data, as compared to that generated by national DHS, is that the MNHR results provide a long-term picture of patterns of ANC access and quality of coverage over time, whereas large national surveys provide limited snapshots.

## Conclusions and implications

Comprehensive ANC confers both independent and collective benefits for mothers and babies. When incorporated as part of an integrated continuum of MNCH services which also includes skilled birth attendance and postpartum care, ANC coverage can contribute to reduction in rates of stillbirth, maternal, and newborn mortality [68]. It is crucial, therefore, to identify gaps in access and coverage, in order to develop targeted strategies by which to ameliorate them.

Results from our study confirm that a crucial barrier to implementation of comprehensive ANC in our Network, particularly at sites in sub-Saharan Africa and Pakistan, is late initiation of care. Thus, in order to increase rates of early initiation of ANC and improve the potential for coverage of key interventions, stakeholders may want to consider implementing strategies for active pregnancy case-finding and explore avenues by which to achieve early, persistent linkage of pregnant women to timely and frequent ANC.

This is one of the largest prospective studies of which we know in which data were collected from a diverse number of global regions for multiple indicators related to the quantity and quality of ANC coverage. By assessing the patterns of ANC and coverage of selected key ANC indicators among a large, population-based sample of 269,710 women over 4 years, this study contributes important information regarding the quantity and quality of ANC services that women receive in rural regions of Africa, Asia, and Latin America. Our results provide investigators with high-quality observational data by which to further explore ANC in their own settings. We have identified key gaps for which stakeholders can develop targeted interventions by which to improve women’s access to an integrated continuum of respectful, comprehensive maternal-newborn and reproductive health services.

## List of abbreviations used

ANC: Antenatal Care; FANC: Focused Antenatal Care; Global Network or GN: Global Network for Women’s and Children’s Health Research; MNCH: Maternal-newborn-child health; MNHR: Maternal Newborn Health Registry; TBAs: Traditional Birth Attendants; WHO: World Health Organization

## Competing interests

The authors declare that they have no competing interests.

## Authors’ contributions

SB conceived of the study, was the primary author, drafted the original manuscript. IM, CT, FE and EL participated in writing and editing the manuscript. CC and KO manage logistics and data collection in Kenya. EAL, FE, AP, SSG, BK, AG, EC, FA, MB, OP, PLH, RJD, KMH, NFK, WAC, RLG, EMM, SS and MKT developed the protocol and participated in site study monitoring and implementation. JM and DDW performed data analyses and data monitoring. All authors reviewed and approved the manuscript prior to publication.

## Peer review

Reviewer reports for this article can be found in Additional file 1.

## Supplementary Material

Additional file 1 Click here for file

## Declarations

This article has been published as part of *Reproductive Health* Volume 12 Supplement 2, 2015: Research reports from the NICHD Global Network for Women’s and Children’s Health Research Maternal and Newborn Health Registry. The full contents of the supplement are available online at http://www.reproductive-health-journal.com/supplements/12/S2. Publication of this supplement was supported by grants from the Eunice Kennedy Shriver National Institute of Child Health and Human Development to RTI International.
