# Supplementary material for: A prospective observational description of frequency and timing of antenatal care attendance and coverage of selected interventions from sites in Argentina, Guatemala, India, Kenya, Pakistan and Zambia
Source: Reprod Health. 2015 Jun 8;12(Suppl 2):S12. doi: 10.1186/1742-4755-12-S2-S12 (PMC4464209; doi:10.1186/1742-4755-12-S2-S12)
Supplement: Additional file 1 [file 1742-4755-12-S2-S12-S1.pdf]

Referee's comments to the authors– this sheet WILL be seen by the author(s) and published with the article

Title	A prospective observational description of frequency and timing of antenatal care attendance and coverage of selected interventions in Argentina, Guatemala, India, Kenya, Pakistan and Zambia
Author(s)	Sherri Bucher, Irene Marete, Constance Tenge, Edward A Liechty, Fabian Esamai, Archana Patel, Shivaprasad S Goudar, Bhalchandra Kodkany, Ana Garces, Elwyn Chomba, Fernando Althabe, Mabel Barreuta, Omrana Pasha, Patricia Hibberd, Richard J Derman, Kevin Otieno, Michael Hambidge, Nancy F Krebs, Waldemar Carlo, Carolyne Chemweno, Robert L Goldenberg, Elizabeth M McClure, Janet Moore, Dennis D Wallace, Sarah Saleem, Marion Koso-Thomas
Referee's name	Carla Bann

When assessing the work, please consider the following points, where applicable:

1. Is the question posed by the authors new and well defined?
2. Are the methods appropriate and well described, and are sufficient details provided to replicate the work?
3. Are the data sound and well controlled?
4. Does the manuscript adhere to the relevant standards for reporting and data deposition?
5. Are the discussion and conclusions well balanced and adequately supported by the data?
6. Do the title and abstract accurately convey what has been found?
7. Is the writing acceptable?

Please make your report as constructive and detailed as possible in your comments so that authors have the opportunity to overcome any serious deficiencies that you find and please also divide your comments into the following categories:

- Major Compulsory Revisions (which the author must respond to before a decision on publication can be reached)
- Minor Essential Revisions (such as missing labels on figures, or the wrong use of a term, which the author can be trusted to correct)
- Discretionary Revisions (which are recommendations for improvement but which the author can choose to ignore)

Where possible please supply references to substantiate your comments.

When referring to the manuscript please provide specific page and paragraph citations where appropriate.

General Comments:
This well-written manuscript provides valuable information on the actual, rather than simply recommended, antenatal care received by women in several low to low-middle income countries. Minor changes to the manuscript would help improve the conveyance of the results to the audience.

Major Compulsory Revisions:

(1) As mentioned in the last paragraph on page 5, a subset of the participants in this study was also included in a trial on emergency obstetric and neonatal care. Additional information is needed about the trial, including how the participants were selected. In particular, it would be helpful to know if there is any reason to expect that antenatal care experiences would have been different among those participants than the remaining ones who were not included in the trial and if so, how results vary with or without the inclusion of those participants.

(continue on the next sheet)

Continued:
Minor Essential Revisions:

(1) The description of the creation of the composite measure on page 10 is somewhat unclear. How do the 1-7 rankings relate to the numbers by category shown in Figure 4? Are the values the number of indicators for which the region had a good, medium, or poor ranking? If so, why was this method chosen rather than another scoring method, such as averaging the rankings? Please clarify in the description of the composite construction and in the figure.

(2) In addition to the overall composite rankings, it would be helpful to present results on rankings for each of the four categories included in the composite listed on page 10: (1) ANC frequency and timing of initiation, (2) preventative interventions, and (3) screening interventions, and (4) birth preparedness. This information would be helpful for identifying specific areas where interventions are needed.

(3) The data appears to be all self-report. Could participants in some of these regions have been tested without their knowledge, such as being aware that they had blood drawn, but not being aware that it was tested for syphilis or HIV?

(4) On page 9, there is a discussion about identifying a method of transport prior to birth. Was this transportation to a location for the delivery?

(5) How does an “antenatal visit” listed in Tables 1 and 2 differ from an “antenatal class” listed in Table 3?

(6) The figure legends refer to five figures; however, there are only four figures included in the manuscript. The flow diagram of the study population (listed as Figure 1 in the figure legends on page 17) was not provided in the manuscript and should be added.

(7) The second paragraph on page 10 refers to a Figure 5; this should be Figure 4.

(8) Figure 1: This figure is not needed because this information is included in Table 1; if desired, it could also be reported in the text.

(9) Figures 3 and 4: These figures are very difficult to read when printed in black and white. Different shading and/or patterns could help improve their readability. In addition, it would be helpful to include the percentages for each category on the bars themselves. Also, if possible for Figure 3, please include a label under each set of countries/sites to indicate the region (e.g., Africa under Kenya and Zambia).

Continued:

Discretionary Revisions:

(1) Given the authors have four years of data, it would be interesting to know if there were any changes in provision of antenatal care over time. In addition, it would be useful to know if there were changes in country or region-level policies during this time period that may have an impact on the types and quality of antenatal care provided.

(2) Table 1: Some of the rows (e.g., Trimester for first ANC visit) indicate N and %; however, only the N is shown. Because the table shows only frequencies and percentages, the table could be made a little clearer by moving the N (%) notation to the top of the column and then including N= on the rows showing the sample sizes (e.g., the first entry for women with delivery could be N=35,660).

(3) Table 2: The top row of data “At least one ANC visit” can be removed because this information is included in Table 1.

Referee's comments to the authors– this sheet WILL be seen by the author(s) and published with the article

Title	A prospective observational description of frequency and timing of antenatal care attendance and coverage of selected interventions in Argentina, Guatemala, India, Kenya, Pakistan and Zambia
Author(s)	Sherri Bucher, Irene Marete, Constance Tenge, Edward A Liechty, Fabian Esamai, Archana Patel, Shivaprasad S Goudar, Bhalchandra Kodkany, Ana Garces, Elwyn Chomba, Fernando Althabe, Mabel Barreuta, Omrana Pasha, Patricia Hibberd, Richard J Derman, Kevin Otieno, Michael Hambidge, Nancy F Krebs, Waldemar Carlo, Carolyne Chemweno, Robert L Goldenberg, Elizabeth M McClure, Janet Moore, Dennis D Wallace, Sarah Saleem, Marion Koso-Thomas
Referee's name	Donald Dudley

When assessing the work, please consider the following points, where applicable:

1. Is the question posed by the authors new and well defined?
2. Are the methods appropriate and well described, and are sufficient details provided to replicate the work?
3. Are the data sound and well controlled?
4. Does the manuscript adhere to the relevant standards for reporting and data deposition?
5. Are the discussion and conclusions well balanced and adequately supported by the data?
6. Do the title and abstract accurately convey what has been found?
7. Is the writing acceptable?

Please make your report as constructive and detailed as possible in your comments so that authors have the opportunity to overcome any serious deficiencies that you find and please also divide your comments into the following categories:

- Major Compulsory Revisions (which the author must respond to before a decision on publication can be reached)
- Minor Essential Revisions (such as missing labels on figures, or the wrong use of a term, which the author can be trusted to correct)
- Discretionary Revisions (which are recommendations for improvement but which the author can choose to ignore)

Where possible please supply references to substantiate your comments.

When referring to the manuscript please provide specific page and paragraph citations where appropriate.

General comments:

1. The authors comment on the main problem with the work, and that has to do with identifying the reasons for the differences between sites. In a way, each site is natural experiment in the provision of prenatal care. Can the authors note utilize the different political and social climates of each site to make some speculation here?

Major compulsory revisions:

Minor Revisions:

2. Minor point: Page 2, methods section of abstract, "ANC" is used but not defined.

3. The authors make a number of assumptions that may not necessarily be based on the strong evidence (e.g. that the number of visits should be 4 or more). Could they provide a bit more background to support these assumptions?

4. Page 8, top of page: Please specify that these are tetanus toxoid "vaccinations" and not vaccines, meaning that women receive these vaccinations during pregnancy, correct?

(continue on the next sheet)

Continued:

5. Page 9, middle of page: The authors note that “70% of women overall identified a method of transport prior to birth”. What does this mean? What do the authors mean by “method of transport”? Does this mean that they have a car, or cart? Please specify.

6. Figure 2 is redundant and could be deleted.

Supplement Editor's comments

Please give in the abstract the rationale or the justification of why you did such analysis.

One relevant contribution of your article is that you are providing information about communities in various countries. However for the reader of your article it is not clear enough, since all over the article you mention the sites as countries. Probably it would be more useful to the reader throughout all the text to qualify what population of these countries is addressed and not the entire country, i.e. in these "communities of India" or similar.

Please provide a better legend for figures. Each figure and it legend should be self-explicative.
